# Association between sleep disturbance in Alzheimer’s disease patients and burden on and health status of their caregivers

**DOI:** 10.1007/s00415-019-09286-0

**Published:** 2019-04-09

**Authors:** Shoki Okuda, Jumpei Tetsuka, Kenichi Takahashi, Yasuo Toda, Takekazu Kubo, Shigeru Tokita

**Affiliations:** 1 Medical Affairs, MSD K.K., Kitanomaru Square, 1-13-12 Kudan-kita, Chiyoda-ku, Tokyo, 102-8667 Japan; 2 Japan Development, MSD K.K., Kitanomaru Square, 1-13-12 Kudan-kita, Chiyoda-ku, Tokyo, 102-8667 Japan

**Keywords:** Alzheimer’s disease, Caregiver burden, Health outcomes, Insomnia, Sleep disturbance, Web-based survey

## Abstract

**Background:**

Sleep disturbance in Alzheimer’s disease (AD) patients may have a negative impact not only on patients themselves but also on the physical and mental health of their caregivers. Detailed analysis of these issues is lacking.

**Objective:**

This study investigated the association between sleep disturbance in AD patients and the burden on, and health status of, their caregivers in Japan.

**Methods:**

We conducted a cross-sectional web-based questionnaire survey among caregivers of AD patients with insomnia symptoms in Japan. Demographic data and Sleep Disorders Inventory (SDI) scores for patients, caregiver burden (Burden Index of Caregivers-11 [BIC-11]) and health status, including Pittsburgh Sleep Quality Index, Patient Health Questionnaire-9, and 12-Item Short Form Health Survey v2, were collected. Multivariate analysis was used to examine the association between the burden and health status of caregivers and sleep disturbance in their care recipients with AD.

**Results:**

A total of 496 caregivers of AD patients with insomnia symptoms were examined in this study. We found that the BIC-11 total score increased as the SDI score increased, indicating a significant positive association, even after adjusting for confounding factors. We also found an association between sleep disturbances of AD patients and health of caregivers (sleep quality, depression, and physical/mental quality of life).

**Conclusion:**

This study demonstrated that sleep disturbance in AD patients was associated with an increased burden and poorer health status of caregivers. Our findings highlight the importance of sleep management in AD patients.

**Electronic supplementary material:**

The online version of this article (10.1007/s00415-019-09286-0) contains supplementary material, which is available to authorized users.

## Introduction

Alzheimer’s disease (AD) is the most common cause of dementia, affecting around 50 million people worldwide [1]. In Japan, the prevalence of AD in people aged 65 years or older is approximately 2% [2]. Symptoms of AD can be divided into core symptoms, comprising mainly cognitive deficits, and behavioral and psychological symptoms of dementia (BPSD). BPSD emerge in the course of disease progression, and can include verbal abuse/violence, depression, hallucination, delusion, delirium, wandering, and sleep disturbance. The greater burden on caregivers stems from BPSD rather than from the core symptoms [3].

Sleep disturbance is categorized as BPSD, with a high prevalence in AD patients of 45–53% [4, 5]. Sleep disturbance in AD patients is characterized by nocturnal insomnia symptoms, such as delays in sleep onset time, decrease in slow wave sleep, increases in nocturnal awakening, and resultant day time sleepiness with increases in daytime napping [6]. The etiology of insomnia in AD patients has not been elucidated. One hypothesis is that sleep architecture in AD patients is markedly disturbed by neurodegeneration and decreased neuron number in the suprachiasmatic nucleus regulating circadian rhythm, or by extensive organic dysfunction of the neurotransmitter system involved in sleep regulation [6, 7].

It is expected that nocturnal BPSD, especially sleep disturbance, not only have a negative impact on patients themselves but also increase the mental and physical burden of their caregivers. In fact, it is reported that insomnia symptoms in AD have a large negative impact on patients’ mental and physical health [8] and that approximately two-thirds of older adult caregivers have some form of sleep disturbance [9]. Nocturnal awakening and subsequent wandering of AD patients cause psychological and physiological stress on family and caregivers [10], leading to institutionalization of patients, such as nursing home placement [11]. In addition, a strong association between sleep disturbance and other BPSD was observed in the very early stages of AD, indicating the important role of sleep in BPSD [12]. Therefore, appropriate management of nighttime sleep of AD patients is important not only for the patients but also to decrease the burden on and improve the health of caregivers. However, such a management has been still unconfirmed and evidence regarding the efficacy of medication against sleep disturbance in AD patients is limited.

Studies of caregiver burden of AD patients have been conducted worldwide [13–18]. The majority of studies have explored the impact of BPSD on caregiver burden [15, 18]. A systematic review of studies found 13 articles examining the association between individual symptoms of BPSD and caregiver burden [15]. Each type of BPSD was shown to have a different relationship to caregiver burden, suggesting that more specific strategies for respective BPSD type are needed [15]. The findings also indicated that sleep disturbance is one of the symptoms most strongly associated with caregiver burden, in addition to irritability and agitation [15].

To our knowledge, few previous studies have been carried out to evaluate the correlation between caregiver burden and patient sleep in AD. A few studies have focused specifically on the impact of sleep disturbance in patients with dementia on caregiver burden in China [19] and US [20]. These cross-sectional studies, however, were relatively small (*N* = 105–130) and did not evaluate caregiver’s sleep quality as one of their health status. The primary objective of this study was to investigate the association between sleep disturbance in Japanese AD patients and associated caregiver burden using a larger sample size (*N* = 500) to perform a multivariate analysis adjusted for potentially confounding factors. The secondary objective of this study was to investigate the association between sleep disturbance in AD patients and the health status of their caregivers.

## Materials and methods

### Study design and participants

This study was a cross-sectional web-based questionnaire survey of caregivers of AD patients. Participants were selected from approximately 5,000,000 Japanese registered with Macromill Carenet, Inc. Informed consent was obtained from adult candidates, who were also asked to respond to a screening questionnaire. Those who did not meet the selection criteria or who met the exclusion criteria were discontinued from the study. Eligible participants were asked to complete all items of the self-reported questionnaire regarding themselves and their care recipients. The target sample size was set at 500 (no formal statistical test was conducted).

Selection criteria of caregiver participants included being 20 years or older, living in Japan, caring for patients presenting with both insomnia symptoms and mild to moderate AD (as evaluated by the caregiver), living with the patient, and being a primary caregiver if caregiving is shared. The caregiver participants were asked to select the appropriate severity about their care recipients from the below three descriptions; Mild (He/She is sometimes forgetful, and often loses things but can still carry out most of activities of daily living on his/her own), Moderate (He/She is very forgetful, gets confused and moody, and needs assistance for many of activities of daily living), Severe (He/She loses mental and physical ability. Assistance for activities of daily living is required 24 h/day). These descriptions were prepared with reference to CDR (Clinical Dementia Rating) and the diagnostic guidelines, so that general people can easily understand. Exclusion criteria included being a caregiver of two or more care recipients; having been diagnosed with schizophrenia, depression, or bipolar disorder; caring for a patient with a mental disorder or mental retardation, Lewy body dementia, frontotemporal dementia, Huntington’s disease, or Parkinson’s disease; caring for a patient with sleep apnea who uses continuous positive airway pressure or bilevel positive airway pressure; and caring for a patient with a physical disability (e.g., loss or impairment of a limb [including fracture], vision disorder, or hearing loss due to injury or congenital abnormality).

Any written information, including the informed consent form, provided to the subject received Ethical Research Committee’s approval (NPO Clinical Research Promotion Network Japan) in advance. Since the research was conducted through an online survey, respondents’ informed consent was obtained electronically.

### Participant characteristics

Data on caregivers included sex, age, job status, relationship to the patient, annual income, educational level, time spent on informal caregiving per week, time spent by paid caregivers (past 2 weeks), time spent on caregiving at night by family (past 2 weeks), and number of caregivers. Data on patients included sex, age, drugs used for insomnia and AD therapy (past 2 weeks), severity of AD, duration of AD, sleep problems of the patients evaluated by the caregiver, and physician-diagnosed sleep disorders.

### Primary outcome measure

Caregiver burden was measured by the Burden Index of Caregivers-11 (BIC-11). The BIC-11 was developed in Japan to measure the burden on caregivers providing home care. Its reliability and validity have been previously demonstrated [21]. The BIC-11 is a brief measure comprising 11 items. It covers five domains (time-dependent burden, emotional burden, existential burden, physical burden, and service-related burden) and one item on total care burden. Caregivers were asked to rate their degree of burden for the past month on a 5-point scale. The score of each domain ranged from 0 to 8 (the score on total care burden ranged from 0 to 4), and the BIC-11 total score ranged from 0 to 44. Higher scores indicated greater caregiving burden.

### Secondary outcome measure

Secondary outcomes included four health indices of caregivers (sleep quality, depression, physical/mental quality of life [QOL]). Sleep quality was measured using the Japanese version of Pittsburgh Sleep Quality Index (PSQI) [22, 23]. Respondents were asked to answer questions on sleep quality, sleep latency, sleep duration, habitual sleep efficiency, sleep disturbance, use of sleeping medication and daytime dysfunction. The total score ranged from 0 to 21. Higher scores indicated poorer quality of sleep. Depressive symptoms were measured using the Japanese version of Patient Health Questionnaire-9 (PHQ-9) [24, 25]. In this study, we used a questionnaire to assess depressive symptoms that occurred in the past 2 weeks. Respondents were asked to rate each item on a 4-point scale. Higher scores indicated more severe depression (range 0–27), and a score of 10 or above indicated probable major depression. Physical QOL and mental QOL were measured using the Japanese version of the 12-Item Short Form Health Survey (SF-12) v2 [26, 27]. In this study, we used the acute form in which respondents were asked about the previous week. The SF-12 v2 provides two summary scores (physical and mental). Higher scores indicated better QOL (range 0–100).

### Sleep disturbance of patients

Sleep disturbances in AD patients were measured using the Sleep Disorders Inventory (SDI) [28]. The SDI was developed from the NPI [29] that was designed to assess neuropsychiatric symptoms frequently observed in persons with dementia. As there was no standardized Japanese version of the SDI at the time of research, we used relevant items from the Japanese version of NPI, which are commercially available [30]. In the SDI, caregivers are asked to rate the frequency and severity of symptoms of care recipients during the past 2 weeks. The SDI comprises seven items (difficulty falling asleep, getting up during the night, nighttime wandering and pacing, awakening you during the night, waking up at night thinking it is daytime, awakening too early in the morning, and sleeping excessively during the day). SDI score was calculated by the product of the average of frequency ratings and the average of severity ratings. Higher scores indicated more severe symptoms (range 0–12).

### Potential confounders

Potential confounding factors regarding caregiver background were sex, age, job status, relationship to the patient, annual income, educational level, time spent by paid caregivers, time spent on caregiving at night by family, and number of caregivers.

Potential confounding factors regarding patient background were sex, age, use of drugs for insomnia/AD therapy (past 2 weeks), severity of AD, duration of AD, and BPSD. BPSD was measured using the Japanese version of NPI-Q [31, 32]. The NPI-Q comprises 12 scales (hallucinations, delusions, agitation/aggression, dysphoria/depression, anxiety, euphoria/elation, apathy/indifference, disinhibition, irritability/lability, aberrant motor behavior, nighttime behavioral disturbances, and appetite/eating disturbances). Caregivers were asked to rate the frequency and severity of symptoms of care recipients during the past month. Higher scores indicated more severe symptoms (range 0–36).

### Statistical methods

Continuous data were described with summary statistics. Categorical data were presented as frequency and percentage, and missing data were excluded from the denominator. For the primary analysis, a multiple regression analysis was performed using BIC-11 as dependent variables and SDI and all other confounding factors as independent variables. A stepwise model selection method with Akaike information criterion was used in the multivariate regression model with *p* = 0.10 for entering a variable into the model and *p* = 0.10 for removing a variable from the model. SDI was always included in the model. The secondary analysis was performed in the same manner as the primary analysis, using each health index (PSQI, PHQ-9, physical component summary, mental component summary) as dependent variables. In addition, a logistic regression analysis was performed with PHQ-9 (a score of ≥ 10 was treated as an event occurrence) as a dependent variable. Missing values were not imputed in the analysis. Statistical significance was set at *p* < 0.05 (two-sided). The 95% confidence interval was calculated. All statistical analyses were performed using SAS 9.4 (SAS institute Inc., Cary, NC, USA).

## Results

**Fig. 1:**
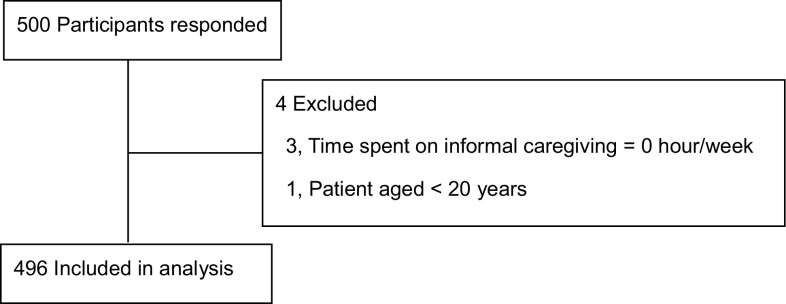
Study flowchart

This survey was conducted between May 15, 2018 and June 18, 2018. Of 3,416,514 candidates, 399,917 responded to the invitation to participate. Eligible responses were obtained from 500 participants (250 men and 250 women). Three participants who spent 0 h/week on informal caregiving and one participant who answered that the care recipient was aged < 20 years were excluded, leaving 496 for analysis (Fig. 1).

### Participant characteristics

AD patients: The proportion of males to females was 27.8% and 72.2%, respectively. The mean age of the total population was 82.8 years [standard deviation (SD) = 9.6], and those aged 75 years or older accounted for 83.9%. Among them, 41.9% were taking sleep aids; 73.8% were taking AD drugs. The severity of AD was mild in 35.7% and moderate in 64.3% and the duration of AD was 4.04 years (SD = 3.21) (Table 1).

**Table 1:** Characteristics of participants

Patient	
Sex, *n* (%)	
Male	138 (27.8)
Female	358 (72.2)
Age, years	
Mean (SD)	82.8 (9.6)
Median (min, max)	84.0 (31, 105)
Category, *n* (%)	
≤ 64	26 (5.2)
65–74	54 (10.9)
≥ 75	416 (83.9)
Sleeping medication, *n* (%)	
Yes	208 (41.9)
No	282 (56.9)
Do not know	6 (1.2)
AD treatment drugs, *n* (%)	
Yes	366 (73.8)
No	120 (24.2)
Do not know	10 (2.0)
Severity of AD, *n* (%)	
Mild	177 (35.7)
Moderate	319 (64.3)
Duration of AD, years	
Mean (SD)	4.04 (3.21)
Median (min, max)	3.30 (0.2, 28.5)
Sleep problems evaluated by caregivers^b^, *n* (%)	
Waking up during night	358 (72.2)
Waking up too early	273 (55.0)
Disturbance of daily rhythm	255 (51.4)
Staying awake too late/past bedtime	255 (51.4)
Abnormal behavior during sleep	164 (33.1)
Sleep disorders diagnosed by physicians^a^, *n* (%)	
Insomnia	151 (30.4)
Circadian rhythm sleep disorder	110 (22.2)
Parasomnia	39 (7.9)
Narcolepsy	27 (5.4)
Sleep apnea	27 (5.4)
Other sleep difficulties	27 (5.4)
Nothing	245 (49.4)

Caregiver	
Sex, *n* (%)	
Male	247 (49.8)
Female	249 (50.2)
Age, years	
Mean (SD)	50.4 (12.4)
Median (min, max)	52.0 (20, 87)
Category, *n* (%)	
20–39	105 (21.2)
40–64	345 (69.6)
65–74	33 (6.7)
≥75	13 (2.6)
Job status, *n* (%)	
Company employee	197 (39.7)
Homemaker	81 (16.3)
Part-timer	60 (12.1)
Not employed	53 (10.7)
Management/executive	19 (3.8)
Public servant	18 (3.6)
Freelance	13 (2.6)
Other	14 (2.8)
Relationship to the patient, *n* (%)	
Son/daughter	403 (81.3)
Husband/wife	24 (4.8)
Grandchild	21 (4.2)
Son-in-law/daughter-in-law	16 (3.2)
Others	32 (6.5)
Annual income, JPY million, *n* (%)	
< 2	39 (7.9)
2 to < 4	87 (17.5)
4 to < 6	101 (20.4)
6 to < 8	76 (15.3)
8 to < 10	49 (9.9)
10 to < 12	35 (7.1)
12 to < 15	13 (2.6)
15 to < 20	8 (1.6)
≥ 20	12 (2.4)
Do not know	25 (5.0)
I choose not to answer	51 (10.3)
Education level, *n* (%)	
Less than high school	15 (3.0)
High school or equivalent	167 (33.7)
Some college, but no degree	13 (2.6)
Two-year degree	95 (19.2)
College graduate	191 (38.5)
Graduate school	15 (3.0)
Time spent on informal caregiving, hours/week	
Mean (SD)	44.5 (39.7)
Time spent by paid caregivers (past 2 weeks)	
Mean (SD)	16.2 (30.6)
Median (min, max)	3.5 (0, 300)
Time spent on caregiving at night by family (past 2 weeks)	
Mean (SD)	8.8 (18.6)
Median (min, max)	0.0 (0, 150)
Number of caregivers, *n* (%)	
Single	209 (42.1)
Multiple	287 (57.9)

496 participants were included in the analysis

*AD* Alzheimer’s disease

^a^Multiple answers were allowed

Caregivers: The proportion of male to female was 49.8% and 50.2%, respectively. The mean age of the total population was 50.4 years (SD = 12.4). Caregivers aged 20–39 years and 40–64 years accounted for 21.2% and 69.6% of participants, respectively. The most common relationship to the patient was daughter or son (81.3%). The mean time spent on caregiving by paid caregivers in the past 2 weeks was 16.2 h (SD = 30.6). The mean time spent on caregiving at night by family in the past 2 weeks was 8.8 h (SD = 18.6) (Table 1).

### Sleep disturbances of AD patients

AD patients showed all the symptoms of sleep disturbance shown in Table 1, and the most common sleep problem recognized by caregivers was nighttime awakening (72.2%). Physicians had diagnosed 30.4% of the patients with insomnia. No diagnosis (“nothing”) accounted for 49.4% of patients (Table 1). The mean SDI score was 1.94 (SD = 2.13) (Table 2). The most frequently reported sleep symptoms in the SDI were getting up during the night (84.3%), sleeping excessively during the day (76.8%), difficulty falling asleep (69.0%), awakening too early in the morning (67.5%), and awakening you during the night (50.8%). The least frequent symptom was present in more than 40% (waking up at night thinking it is daytime, 41.3%). A higher percentage (6.9–9.5%) of AD patients, who were getting up during the night, sleeping excessively during the day, and awakening too early in the morning, were rated “marked” severity compared with those who had other sleep disturbance symptoms.

**Table 2:** Association of sleep disturbances of AD patients with caregiver burden (BIC-11 total) in a multiple linear regression model

Variable	Outcome (score range)	Mean (SD)	Coefficient (95% CI)	*p* value
Patient	SDI^a^ (0–12)	1.94 (2.13)	1.18 (0.80, 1.56)	< 0.001
	Sex (Male/Female^b^)	–	1.99 (0.46, 3.52)	0.011
	NPI-Q^c^ (0–36)	9.6 (6.7)	0.26 (0.14, 0.39)	< 0.001
Caregiver	Time spent on caregiving at night by family (hr)	8.8 (18.6)	0.05 (0.01, 0.09)	0.010
	Number of caregivers (Single/Multiple^b^)	–	1.20 (− 0.19, 2.60)	0.091

Confounding factors except for SDI were selected in a stepwise manner

*BIC* Burden Index of Caregivers-11, *CI* confidence interval, *NPI-Q* Neuropsychiatric Inventory-Brief Questionnaire Form, *SDI* Sleep Disorders Inventory

^a^Higher scores indicate more severe sleep disturbances

^b^Reference

^c^Higher scores indicate more severe behavioral and psychological symptoms

### Behavioral and psychological symptoms in AD patients

The mean NPI-Q score was 9.6 (SD = 6.7) (Table 2). The most frequent symptoms were apathy/indifference (70.6%), nighttime behavior (70.4%), irritability/lability (55.4%), agitation/aggression (52.6%), and aberrant motor behaviors (50.6%). The least frequent symptom, hallucinations, was present in nearly one-third of patients (32.5%). Symptoms rated as severe in at least 5% of AD patients were irritability/lability and nighttime behavior.

### Health-related parameters in caregivers

The mean BIC-11 total score in caregivers of AD patients was 22.7 (maximum score 44) (Table 3), suggesting that they perceived a moderate to severe burden. The mean PSQI score was 8.1, indicating poor sleep quality. The mean PHQ-9 score was 8.4, indicating mild depressive symptoms. The mean physical and mental component summary (QOL) were 47.31 and 45.99, respectively (Table 4).

**Table 3:** Association of sleep disturbances of AD patients with their caregiver burden (BIC-11 total and domain scores) in multiple linear regression model

Outcome (score range)	Mean (SD)	Coefficient (95% CI) for SDI	*p* value
BIC total^a^ (0–44)	22.7 (8.8)	1.18 (0.80, 1.56)	< 0.001
Time-dependent burden (0–8)^b^	5.1 (1.9)	0.26 (0.19, 0.33)	< 0.001
Emotional burden (0–8)^c^	4.4 (2.0)	0.18 (0.09, 0.28)	< 0.001
Existential burden (0–8)^d^	4.2 (2.0)	0.17 (0.08, 0.26)	< 0.001
Physical burden (0–8)^e^	3.4 (2.1)	0.25 (0.16, 0.35)	< 0.001
Service-related burden (0–8)^f^	3.4 (2.0)	0.24 (0.14, 0.33)	< 0.001
Total care burden (0–4)^g^	2.2 (1.0)	0.11 (0.06, 0.16)	< 0.001

Confounding factors except for SDI were selected in a stepwise manner

*BIC* Burden Index of Caregivers-11, *CI* confidence interval, *SDI* Sleep Disorders Inventory

^a^Higher scores indicate greater caregiver burden

^b^Two variables (severity of disease and number of caregivers) were selected

^c^Three variables (Neuropsychiatric Inventory-Brief Questionnaire Form [NPI-Q], sex of patients, and time spent on caregiving at night by family) were selected

^d^Five variables (NPI-Q, sex of patients, AD treatment drugs, job status, and time spent on caregiving at night by family) were selected

^e^Seven variables (NPI-Q, sex of patients, sleeping medication, AD treatment drugs, time spent by paid caregivers, time spent on caregiving at night by family, and number of caregivers) were selected

^f^Two variables (NPI-Q and sex of patients) were selected

^g^Two variables (NPI-Q and relationship to the patient) were selected

**Table 4:** Association of sleep disturbances of AD patients with caregiver health outcomes in a multiple linear regression model

Outcome (score range)	Mean (SD)	Coefficient (95% CI) for SDI	*p* value
PSQI^a,b^ (0–21)	8.1 (3.6)	0.62 (0.46, 0.78)	< 0.001
PHQ-9^c,d^ (0–27)	8.4 (6.2)	1.03 (0.77, 1.29)	< 0.001
Physical component summary^e,f^ (0–100)	47.31 (13.20)	− 1.30 (− 1.84, − 0.77)	< 0.001
Mental component summary^e,g^ (0–100)	45.99 (10.16)	− 0.84 (− 1.26, − 0.43)	< 0.001

Confounding factors except for SDI were selected in a stepwise manner

*CI* confidence interval, *PHQ-9* patient health questionnaire-9, *PSQI* Pittsburgh sleep quality index, *SDI* Sleep Disorders Inventory

^a^486 participants were included in the analysis. Higher scores indicate poorer quality of sleep

^b^Four variables (Neuropsychiatric Inventory-Brief Questionnaire Form [NPI-Q], sleeping medication, AD treatment drugs, and number of caregivers) were selected

^c^Higher scores indicate more severe depression

^d^Continuous variables are shown. Six variables (NPI-Q, sex of patients, AD treatment drugs, sex of caregivers, age of caregivers, and job status) were selected. Logistic regression analysis using PHQ-9 (dichotomous value) also showed a significant positive association between SDI and PHQ-9 (odds ratio for SDI 1.35; 95% CI 1.20–1.54; *p* < 0.001)

^e^Calculated using the 12-Item Short Form Health Survey v2. Higher scores indicate better quality of life

^f^Five variables (sex of patients, sleeping medication, age of caregivers, time spent by paid caregivers, and time spent on nighttime caregiving by family) were selected

^g^One variable (job status) was selected

### Association between sleep disturbances of patients and caregiver burden and health

Tables 2 and 3 show the association between sleep disturbance in AD patients and caregiver burden, plus confounding factors in a multiple linear regression model (details are shown in Supplementary Tables 1, 2). Using the BIC-11 total score as a dependent variable, SDI score and four variables (NPI-Q, sex of patients, time spent on caregiving at night by family, and number of caregivers) were selected. The BIC-11 total score increased as the SDI score increased, indicating a significant positive association (Table 2). The same pattern was observed in Table 3 for time dependent, emotional, existential, physical, service-related, and total care burdens. As the SDI increased, each burden included in the analysis also increased.

The association between sleep disturbance in AD patients and caregiver health was similarly demonstrated in Table 4 (details are shown in Supplementary Tables 3–6). The PSQI score was used as a dependent variable, the PSQI score increased as the SDI score increased, indicating a significant positive association. The PHQ-9 score (a continuous variable) was used as a dependent variable, the PHQ-9 score increased as the SDI score increased, indicating a significant positive association. Using the physical component summary score and mental component summary score as a dependent variable in each model, each variable decreased as the SDI score increased, indicating a significant negative association.

## Discussion

This study focused on sleep disturbances in AD patients from the caregivers’ perspective, with regard to caregiver burdens and their health status (sleep quality, depression, physical QOL, and mental QOL). The results of this study showed that sleep disturbances in AD patients were associated with an increased burden and poorer health status of caregivers, suggesting the importance of deepening our understanding of sleep disturbances in AD patients.

The mean age of AD patients in this study was 82.8 years, and those aged 75 years or older accounted for more than 80%. The male-to-female ratio was 3:7. This result was similar to a previous survey of AD patients in the National Health and Wellness Survey (NHWS) in Japan [33]. The proportion of patients aged 75 years or older in this study was also similar to that of care recipients in a nationwide survey conducted by the Ministry of Health, Labour and Welfare (MHLW) [34]. Sleep disturbance is one of the neurobehavioral characteristics frequently observed in AD patients. Approximately half of AD patients have previously been reported to suffer from insomnia [4, 5]. In this study, insomnia was the most prevalent sleep disorder diagnosed by physicians. AD patients typically complain about nocturnal sleep fragmentation and nocturnal awakening, early awakening, delayed sleep onset, decreased deep sleep, and increased daytime napping due to insufficient sleep at night [6]. We found that a high percentage of AD patients showed frequent and severe insomnia symptoms such as getting up during the night, sleeping excessively during the day, and awakening too early in the morning, as assessed by SDI. The frequency of BPSD reported in this study, as assessed by NPI-Q, was also similar to the results of previous studies; five symptoms (apathy, sleep abnormality, irritability, agitation, and abnormal behavior) were observed in at least 50% of AD patients [31, 32].

The mean age of the caregivers was 50.4 years, and more than half of them were aged 40–64 years. The proportion of male to female caregivers was similar. These caregiver characteristics corroborated the findings of previous studies demonstrating the burden of caregivers of AD patients in NHWS [33]. The most common relationship of caregivers to AD patients was child (81.3%), whereas only 4.8% of caregivers were spouses. In the nationwide survey by MHLW, the majority of the primary caregivers were women who lived with the patients and the most frequent age group of caregivers was 60–69 years in both males and females [34]. The proportion of child caregivers and that of spousal caregivers was similar [34]. The distribution of caregivers of AD patients in this study was slightly skewed toward males and children of patients compared with the nationwide survey by MHLW. Based on the results of the NHWS study and the present results, caregivers of patients with AD may be characterized as follows: a similar proportion of males and females, relatively younger than the general caregiver population, and the majority are children taking care of their parents. These characteristics, however, might be dependent on the participants registered to this web panel, thus these findings cannot be generalized.

Caregivers in this study showed decreased sleep quality. This result was consistent with findings of Montgomery and colleagues, who demonstrated that insomnia was a common comorbidity in caregivers [33]. In addition, a perception of burden and depressive symptoms were also observed. These findings suggest that the caregivers of AD patients in this study have some physical and mental problems.

This study showed that sleep disturbances in AD patients were associated with all domains of burden and all four health indices in their caregivers, even after adjusting BPSD and other factors. Majority of caregivers in our study were sons/daughters of their care recipients, suggesting that sleep disturbances in AD patients have a bigger negative impact on the burden and health of younger caregivers compared with other groups of aged caregivers (i.e., spouses of care recipients), who have age-related deterioration in physical condition. In this study, however, the number of the spouse caregiver was limited, thus we could not analyze the association between the relationship to the patients and other parameters. One study shows that spousal caregivers showed significantly less burden than younger caregivers (those caring for their parent) [35]. Younger caregivers are often balancing nursing care (for their mother or father), child rearing and more likely, their job. Thus, caregiving while working may increase their burden.

We examined associations of SDI with caregivers’ burden and health, but there is a possibility that the relationship between caregiver burden and health depends on the type of sleep disturbances. For example, even a low frequency of nocturnal wandering would be expected to generate high caregiver distress; in contrast, caregiver distress associated with a high frequency of daytime napping has been shown to be low [28]. In this study, some caregivers answered in the questionnaire and SDI that their care recipients showed abnormal behaviors (for example, wandering, pacing, or planning to go out, etc.) during the night, suggesting that such behaviors might influence the result of this study. However, a detailed analysis was not carried out in this study. We used NPI-Q (to measure for BPSD) as a confounding factor; however, the factors that aggravate caregivers’ physical and mental status may be a combination of specific sleep disturbances and the type of BPSD, such as nocturnal awakening and delirium.

We collected data from the caregivers who were caring for mild to moderate AD patients. The severity of more than half of the AD patients was moderate. In a previous study of AD patients using SDI, sex and AD duration were not associated with sleep disturbances, but the severity of AD was associated with the severity of sleep disturbance [28]. In this study, AD severity had no impact on the association between sleep disturbance of AD patients and caregiver burden (except time-dependent burden) or their health status, but the results of this study might have been different if a patient group with a different severity of AD had been used.

Few studies have reported on the influence of pharmacotherapy of AD patients on caregiver burden. Sleeping medication and antipsychotics are used for insomnia in AD patients; however, information on their effect on sleep disturbances and behavioral disorders is limited [7, 36]. Currently, no studies have clearly described the influence of improving insomnia with hypnotics in AD patients on caregiver burden [36]. In this study, the effect of hypnotics in AD patients was not associated with any of the outcomes. This might be because only 41.9% of patients were taking hypnotics and many patients without sleeping medication had very low SDI scores. Also, the types of hypnotics were not identified in this study. Further investigation is needed to understand the impact of sleep medication of AD patients on caregivers’ burden.

This study has several limitations. Since information on nonparticipants was not available, the influence of differences in characteristics between participants and nonparticipants on the results remains unclear. In addition, since this is a cross-sectional study, the causal relationship cannot be determined. It might be possible, for example, that depression in caregivers influences sleep disturbance in patients. Participants in this study were registrants with an online research company; therefore, those who did not have/could not use a computer or mobile phone were not included. Therefore, the characteristics of the web survey could be one reason for the atypical distribution (the small number of the spouses of the patients) of the age of the caregiver participants in this study. Based on the findings of previous studies, many confounding factors were considered in the present analysis; however, there are still other potential influencing characteristics of patients and caregivers, such as other symptoms directly related to AD (including those seen during the daytime) or their comorbidities (for example, cardiac insufficiency, depression, nocturia, etc), which might have affected the results. In the case of the survey participants who selected ‘multiple caregivers’ for the number of caregivers, there were one or more secondary (informal or professional) caregivers helping them. In this study, we did not ask the information about the secondary caregivers from them, but the degree of their involvement may impact the burden of the primary caregivers. As only the information about the current health status of caregivers was collected in this study, we cannot discuss the influence of their health status prior to taking care of AD patients.

In conclusion, our study showed that sleep disturbance in AD patients was associated with an increased burden and poorer health status of caregivers, suggesting the importance of understanding sleep disturbances in AD patients. This study further revealed that sleep-related symptoms were not diagnosed and the medication for sleep disturbance was not taken in approximately half of AD patients, despite being recognized by the caregivers. This finding may reflect the reality of the current clinical situation, showing that these patients/caregivers may not have discussed their sleep disturbance with their doctors. Deepening the understanding of sleep disturbances in AD patients is an important future goal. Traditionally, sleep disturbances in AD patients have been treated as a part of the BPSD spectrum; however, our results suggest the importance of focusing on sleep disturbances from the perspective of their burden on caregivers and the impact on their health.

## Electronic supplementary material

Below is the link to the electronic supplementary material.

Supplementary material 1 (PDF 162 KB)
