# Supplementary material for: Association between sleep disturbance in Alzheimer’s disease patients and burden on and health status of their caregivers
Source: J Neurol. 2019 Apr 9;266(6):1490–500. doi: 10.1007/s00415-019-09286-0 (PMC6517338; doi:10.1007/s00415-019-09286-0)
Supplement: Supplementary file 1 — Supplementary material 1 (PDF 162 KB) [file 415_2019_9286_MOESM1_ESM.pdf]

Article title:

Association between sleep disturbance in Alzheimer's disease patients and burden on and health status of their caregivers

Journal name:

Journal of Neurology

Authors:

Shoki Okuda (1), Jumpei Tetsuka (1), Kenichi Takahashi (2), Yasuo Toda (1), Takekazu Kubo (1), Shigeru Tokita (1)

Author affiliations and addresses:

(1) Medical Affairs, MSD K.K., Kitanomaru Square, 1-13-12 Kudan-kita, Chiyoda-ku, Tokyo 102-8667, Japan

(2) Japan Development, MSD K.K., Kitanomaru Square, 1-13-12 Kudan-kita, Chiyoda-ku, Tokyo 102-8667, Japan

Corresponding author:

Shoki Okuda

Email: shoki.okuda@merck.com

Table I Association of SDI with BIC-11 in a multiple linear regression model[a]

Variable			Coefficient (95% CI)	*p* value
Patient	SDI		1.18 (0.80, 1.56)	<0.001
	Sex	Female (reference)		
		Male	1.99 (0.46, 3.52)	0.011
	NPI-Q		0.26 (0.14, 0.39)	<0.001
Caregiver	Time spent on caregiving at night by family		0.05 (0.01, 0.09)	0.010
	Number of caregivers	Multiple (reference)		
		Single	1.20 (-0.19, 2.60)	0.091

BIC-11, Burden Index of Caregivers-11; CI, confidence interval; NPI-Q, Neuropsychiatric Inventory-Brief Questionnaire Form; SDI, Sleep Disorders Inventory.

[a] Confounding factors except for SDI were selected in a stepwise manner.

Table II Association of SDI with BIC-11 sub-domains in a multiple linear regression model[a]

Variable			Coefficient (95% CI)	*p* value
		Time-dependent burden		
Patient	SDI		0.26 (0.19, 0.33)	<0.001
	Severity of AD	Mild (reference)		
		Moderate	0.74 (0.41, 1.06)	<0.001
Caregiver	Number of caregivers	Multiple (reference)		
		Single	0.41 (0.10, 0.72)	0.009
		Emotional burden		
Patient	SDI		0.18 (0.09, 0.28)	<0.001
	Sex	Female (reference)		
		Male	0.32 (-0.06, 0.69)	0.097
	NPI-Q		0.05 (0.02, 0.08)	0.001
Caregiver	Time spent on caregiving at night by family		0.01 (0.00, 0.02)	0.060
		Existential burden		
Patient	SDI		0.17 (0.08, 0.26)	<0.001
	Sex	Female (reference)		
		Male	0.37 (-0.01, 0.75)	0.054
	AD treatment drugs	No (reference)		
		Yes	-0.44 (-0.84, -0.05)	0.028
		Do not know	-0.88 (-2.08, 0.32)	0.151
	NPI-Q		0.06 (0.03, 0.09)	<0.001
Caregiver	Job status	Company employee (reference)		
		Public servant	-0.63 (-1.53, 0.28)	0.172
		Management/executive	-1.33 (-2.21, -0.45)	0.003
		Self-employed	0.08 (-0.55, 0.71)	0.804
		Freelance	0.04 (-1.01, 1.09)	0.938
		Homemaker	-0.35 (-0.83, 0.14)	0.161
		Part-timer	-0.42 (-0.97, 0.13)	0.134
		Other	-0.64 (-1.65, 0.37)	0.215

		Not employed	-0.61 (-1.19, -0.04)	0.037
	Time spent on caregiving at night by family		0.01 (0.00, 0.02)	0.066
		Physical burden		
Patient	SDI		0.25 (0.16, 0.35)	<0.001
	Sex	Female (reference)		
		Male	0.67 (0.29, 1.04)	0.001
	Sleeping medication	No (reference)		
		Yes	0.67 (0.31, 1.04)	<0.001
		Do not know	-0.13 (-1.73, 1.47)	0.874
	AD treatment drugs	No (reference)		
		Yes	-0.53 (-0.93, -0.12)	0.011
		Do not know	-0.62 (-1.88, 0.64)	0.332
	NPI-Q		0.04 (0.01, 0.07)	0.016
Caregiver	Time spent by paid caregivers		-0.01 (-0.01, 0.00)	0.055
	Time spent on caregiving at night by family		0.02 (0.01, 0.03)	0.001
	Number of caregivers	Multiple (reference)		
		Single	0.32 (-0.03, 0.66)	0.070
		Service-related burden		
Patient	SDI		0.24 (0.14, 0.33)	<0.001
	Sex	Female (reference)		
		Male	0.57 (0.20, 0.93)	0.002
	NPI-Q		0.06 (0.03, 0.09)	<0.001
		Total care burden		
Patient	SDI		0.11 (0.06, 0.16)	<0.001
	NPI-Q		0.04 (0.03, 0.06)	<0.001
Caregiver	Relationship to the patient	Husband/wife (reference)		
		Son/daughter	0.03 (-0.36, 0.42)	0.884
		Grandchild	-0.27 (-0.83, 0.28)	0.336
		Son-in-low/daughter-	-0.09 (-0.69, 0.52)	0.778

	in-low		
	Others	-0.46 (-0.96, 0.05)	0.075

AD, Alzheimer's disease; BIC-11, Burden Index of Caregivers-11; CI, confidence interval; NPI-Q, Neuropsychiatric Inventory-Brief Questionnaire Form; SDI, Sleep Disorders Inventory.

[a] Confounding factors except for SDI were selected in a stepwise manner.

Table III Association of SDI with PSQI in a multiple linear regression model[a]

Variable			Coefficient (95% CI)	*p* value
Patient	SDI		0.62 (0.46, 0.78)	<0.001
	Sleeping medication	No (reference)		
		Yes	1.12 (0.52, 1.71)	<0.001
		Do not know	2.23 (-0.55, 5.02)	0.116
	AD treatment drugs	No (reference)		
		Yes	-0.99 (-1.65, -0.32)	0.004
		Do not know	-1.23 (-3.37, 0.90)	0.258
	NPI-Q		0.08 (0.03, 0.13)	0.002
Caregiver	Number of caregivers	Multiple (reference)		
		Single	0.57 (0.01, 1.14)	0.047

AD, Alzheimer's disease; CI, confidence interval; NPI-Q, Neuropsychiatric Inventory-Brief Questionnaire Form; PSQI, Pittsburgh Sleep Quality Index; SDI, Sleep Disorders Inventory.

[a] Confounding factors except for SDI were selected in a stepwise manner.

Table IV Association of SDI with PHQ-9 in a multiple linear regression model[a]

Variable			Coefficient (95% CI)	*p* value
Patient	SDI		1.03 (0.77, 1.29)	<0.001
	Sex	Female (reference)		
		Male	1.25 (0.18, 2.32)	0.023
	AD treatment drugs	No (reference)		
		Yes	-1.68 (-2.80, -0.56)	0.003
		Do not know	-2.94 (-6.34, 0.45)	0.089
	NPI-Q		0.21 (0.12, 0.29)	<0.001
Caregiver	Sex	Female (reference)		
		Male	-1.09 (-2.16, -0.02)	0.046
	Age		-0.05 (-0.09, 0.00)	0.034
	Job status	Company employee (reference)		
		Public servant	-1.76 (-4.32, 0.79)	0.176
		Management/executive	-0.72 (-3.19, 1.76)	0.570
		Self-employed	1.52 (-0.32, 3.36)	0.105
		Freelance	1.92 (-1.05, 4.89)	0.204
		Homemaker	-2.30 (-3.82, -0.78)	0.003
		Part-timer	-1.31 (-2.93, 0.31)	0.112
		Other	-1.57 (-4.44, 1.30)	0.284
		Not employed	-0.05 (-1.76, 1.67)	0.956

AD, Alzheimer's disease; CI, confidence interval; NPI-Q, Neuropsychiatric Inventory-Brief Questionnaire Form; PHQ-9, Patient Health Questionnaire-9; SDI, Sleep Disorders Inventory.

[a] Confounding factors except for SDI were selected in a stepwise manner.

Table V Association of SDI with PHQ-9 (point ≥ 10) in a multiple logistic regression model[a]

Variable			Odds ratio (95% CI)	*p* value
Patient	SDI		1.35 (1.20, 1.54)	<0.001
	Sex	Female (reference)		
		Male	1.72 (1.08, 2.73)	0.023
	AD treatment drugs	No (reference)		
		Yes	0.57 (0.35, 0.94)	0.027
		Do not know	0.37 (0.07, 2.04)	0.254
	NPI-Q		1.08 (1.04, 1.12)	<0.001
Caregiver	Age		0.97 (0.95, 0.99)	0.003
	Job status	Company employee (reference)		
		Public servant	0.32 (0.09, 1.07)	0.065
		Management/executive	0.78 (0.26, 2.31)	0.650
		Self-employed	1.42 (0.63, 3.16)	0.395
		Freelance	1.16 (0.31, 4.43)	0.824
		Homemaker	0.49 (0.25, 0.94)	0.033
		Part-timer	0.77 (0.39, 1.54)	0.459
		Other	0.18 (0.04, 0.90)	0.036
		Not employed	1.31 (0.61, 2.78)	0.489

AD, Alzheimer's disease; CI, confidence interval; NPI-Q, Neuropsychiatric Inventory-Brief Questionnaire Form; PHQ-9, Patient Health Questionnaire-9; SDI, Sleep Disorders Inventory.

[a] Confounding factors except for SDI were selected in a stepwise manner.

Table VI Association of SDI with SF-12v2 in a multiple linear regression model[a]

Variable			Coefficient (95% CI)	*p* value
Physical component summary				
Patient	SDI		-1.30 (-1.84, -0.77)	<0.001
	Sex	Female (reference)		
		Male	-2.70 (-5.22, -0.18)	0.035
	Sleeping medication	No (reference)		
		Yes	-3.81 (-6.21, -1.41)	0.002
		Do not know	1.90 (-8.41, 12.20)	0.718
Caregiver	Age		-0.10 (-0.20, -0.01)	0.030
	Time spent by paid caregivers		0.04 (0.01, 0.08)	0.025
	Time spent on caregiving at night by family		-0.09 (-0.16, -0.03)	0.003
Mental component summary				
Patient	SDI		-0.84 (-1.26, -0.43)	<0.001
Caregiver	Job status	Company employee (reference)		
		Public servant	5.28 (0.48, 10.08)	0.031
		Management/executive	2.60 (-2.06, 7.27)	0.273
		Self-employed	-3.61 (-6.95, -0.28)	0.034
		Freelance	-1.31 (-6.88, 4.26)	0.644
		Homemaker	-1.27 (-3.84, 1.29)	0.329
		Part-timer	-1.42 (-4.29, 1.45)	0.333
		Other	-1.88 (-7.25, 3.49)	0.491
		Not employed	-5.44 (-8.45, -2.43)	<0.001

CI, confidence interval; SDI, Sleep Disorders Inventory; SF12 v2, 12-Item Short Form Health Survey v2.

[a] Confounding factors except for SDI were selected in a stepwise manner.
